# P-2266. Prediction of infection and acute rejection by QuantiFERON-Monitor in kidney transplant recipient

**DOI:** 10.1093/ofid/ofae631.2419

**Published:** 2025-01-29

**Authors:** Gisela Serra Rodrigues Costa, José Otto Reusing Junior, Fabiana Otto Agena, Vanessa Vidotto Frade, Elias David-Neto, Ligia Camera Pierrotti

**Affiliations:** HC-FMUSP, São Paulo, Sao Paulo, Brazil; HCFMUSP, São Paulo, Sao Paulo, Brazil; HCFMUSP, São Paulo, Sao Paulo, Brazil; DASA, São Paulo, Sao Paulo, Brazil; HCFMUSP, São Paulo, Sao Paulo, Brazil; HCFMUSP/DASA, São Paulo, Sao Paulo, Brazil

## Abstract

**Background:**

Infection is an important complication after solid organ transplant (Tx), so a proper balance between rejection risk and reliable immune responses remains a challenge. One of approaches to evaluate net state of immunosuppression in Tx recipients is the measure of interferon-ɣ (INF-ɣ) release through QuantiFERON-monitor assay (QFM). This assay measures both innate and acquired immune responses. This study aimed to assess the association of QFM with infection and acute rejection (AR) occurring in the first-year post-Tx in kidney transplant recipients (KTR) cohort.

Box Plot of QFM results according to study timeline

**Figure:**
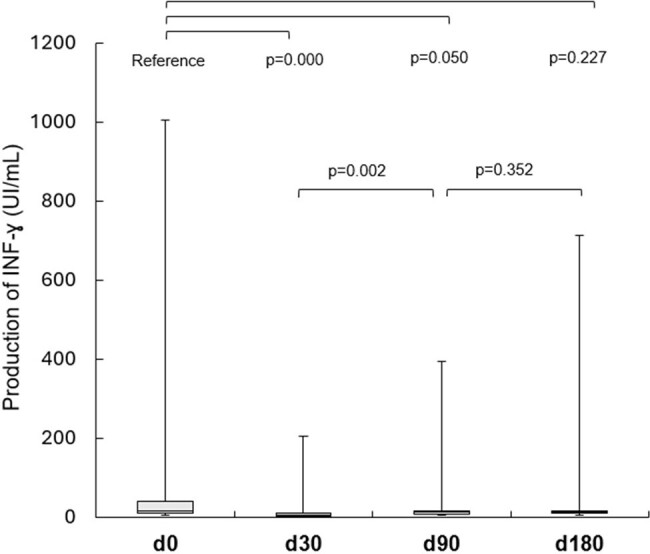

**Methods:**

Prospective cohort study of KTR who were given anti-thymocyte globulin (ATG) induction therapy enrolled in August/2018 to August/2019. QFM tests were collected at the Tx procedure (d0), 30 (d30), 90 (d90) and 180 (d180) days post-Tx and results were presented as medians of INF-ɣ values. Infections were classified in severe (hospitalization), opportunist, bacterial and clinically significant cytomegalovirus events. AR was biopsy proven or defined by treatment with ATG or corticosteroids.

Kinetics Plots of INF-ɣ stratified by Infection episodes and study timeline

**Figure:**
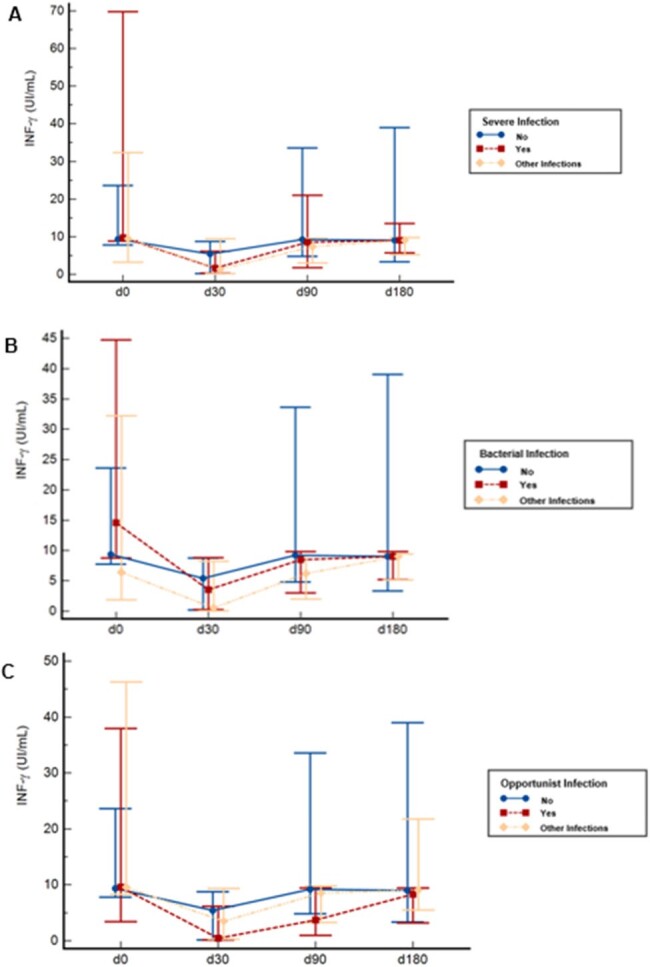

A - Medians and interquartile ranges of INF-ɣ release according to study timeline and Severe Infection. B - Medians of INF-ɣ release according to study timeline and Bacterial Infection and C - Medians of INF-ɣ release according to study timeline and Opportunist Infection

**Results:**

There were 68 KTR, of those 50 had 99 infections, resulting in a cumulative incidence estimated at 73.5% (IC95% 54.6-96.9%) and an incidence density of 4.4 per 1000 Tx-days (CI95% 3.2-5.7). Additionally, there were 16 AR in 16 KTR, with a cumulative incidence of 23.5% (CI95% 95% 13-38%) and an incidence density of 0.77 per 1000 Tx-day (IC95% 0,44- 1,26). The plots depicting INF-ɣ kinetics revealed a significant decline in median values at 30 days post-Tx (p=0,000), followed by a recovery to pre-Tx values at 180 days post-Tx. We found a statistically significant association between QFM results at day 0 and severe infections occurring 30-90 days post-Tx and bacterial infections occurring 30-180 days post-Tx. Furthermore, there was an association between QFM results at d30 and opportunistic infections occurring 30-90 days post-Tx. QFM collected at day 0 also exhibited an association with AR during the first-year post-Tx (p=0,014).

Kinetics Plots of INF-ɣ stratified by Acute Rejection episodes and study timeline

**Figure:**
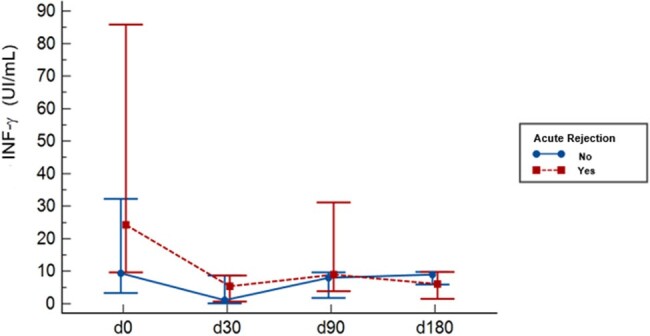

**Conclusion:**

Preliminary results confirmed a high incidence density of infection in this KTR cohort and suggested the potential value of QFM for risk assessment of acute rejection and severe, bacterial, and opportunistic infections in the first-year post-Tx when collected at day 0 and day 30 post-Tx.

**Disclosures:**

All Authors: No reported disclosures

